# Aberrant dynamics of cognitive control and motor circuits predict distinct restricted and repetitive behaviors in children with autism

**DOI:** 10.1038/s41467-021-23822-5

**Published:** 2021-06-10

**Authors:** Kaustubh Supekar, Srikanth Ryali, Percy Mistry, Vinod Menon

**Affiliations:** 1 Department of Psychiatry & Behavioral Sciences, Stanford University, Stanford, CA USA; 2 Department of Neurology & Neurological Sciences, Stanford University, Stanford, CA USA; 3 Stanford Neuroscience Institute, Stanford University, Stanford, CA USA

**Keywords:** Predictive markers, Autism spectrum disorders

## Abstract

Restricted and repetitive behaviors (RRBs) are a defining clinical feature of autism spectrum disorders (ASD). RRBs are highly heterogeneous with variable expression of circumscribed interests (CI), insistence of sameness (IS) and repetitive motor actions (RM), which are major impediments to effective functioning in individuals with ASD; yet, the neurobiological basis of CI, IS and RM is unknown. Here we evaluate a unified functional brain circuit model of RRBs and test the hypothesis that CI and IS are associated with aberrant cognitive control circuit dynamics, whereas RM is associated with aberrant motor circuit dynamics. Using task-free fMRI data from 96 children, we first demonstrate that time-varying cross-network interactions in cognitive control circuit are significantly reduced and inflexible in children with ASD, and predict CI and IS symptoms, but not RM symptoms. Furthermore, we show that time-varying cross-network interactions in motor circuit are significantly greater in children with ASD, and predict RM symptoms, but not CI or IS symptoms. We confirmed these results using cross-validation analyses. Moreover, we show that brain-clinical symptom relations are not detected with time-averaged functional connectivity analysis. Our findings provide neurobiological support for the validity of RRB subtypes and identify dissociable brain circuit dynamics as a candidate biomarker for a key clinical feature of ASD.

## Abstract (web summary)

Restricted and repetitive behaviors (RRBs) are a core clinical feature of autism, yet the brain basis of RRBs is unknown. Here, the authors demonstrate that aberrant cognitive control and motor circuit dynamics differentially predict three distinct symptom clusters that define RRBs.

## Introduction

Restricted and repetitive behaviors (RRBs) have long been recognized as a core symptom of autism spectrum disorders (ASD)^1^. RRBs are the earliest detectable behavioral predictors of ASD and have adverse long-term consequences for acquisition of crucial life skills in individuals with the disorder^2,3^. Critically, recent changes to the Diagnostic and Statistical Manual of Mental Disorders have identified RRBs as central to understanding heterogeneity of clinical presentations in ASD^4^. However, RRBs remain a grossly understudied aspect of ASD research and the underlying brain circuits are unknown^5,6^.

RRBs include behaviors such as preoccupation with objects, ritualized patterns of behavior, highly restricted/fixated interests and stereotyped/repetitive motor (RM) movements^1^. Although RRBs were traditionally defined as a unitary construct^5,7^, there is growing evidence that RRBs are a heterogeneous construct that can be factored into three distinct phenotypic characteristics: circumscribed interests (CI), insistence on sameness (IS), and RM actions^8,9^. CI and IS include adherence to routines and restricted patterns of interest and are thought to be cognitive in nature^10,11^. In contrast, RM includes hand flapping, rocking, and head banging, and are likely to be primarily motoric in origin^10,11^. Recent evidence suggest that clinical phenotypic features may be more tightly linked to dynamical properties of functional brain circuits as they reflect complex nonlinear neural dynamics and fluctuations in internal mental states^12–15^. Whether CI, IS, and RM are associated with distinct dynamic brain circuit properties in ASD is currently unknown. Uncovering the brain circuit mechanisms underlying these heterogeneous RRB symptoms/subtypes is important for a more precise understanding of the neurobiology of ASD and for further validating the distinctiveness of these individual phenotypic constructs.

**Fig. 1:**
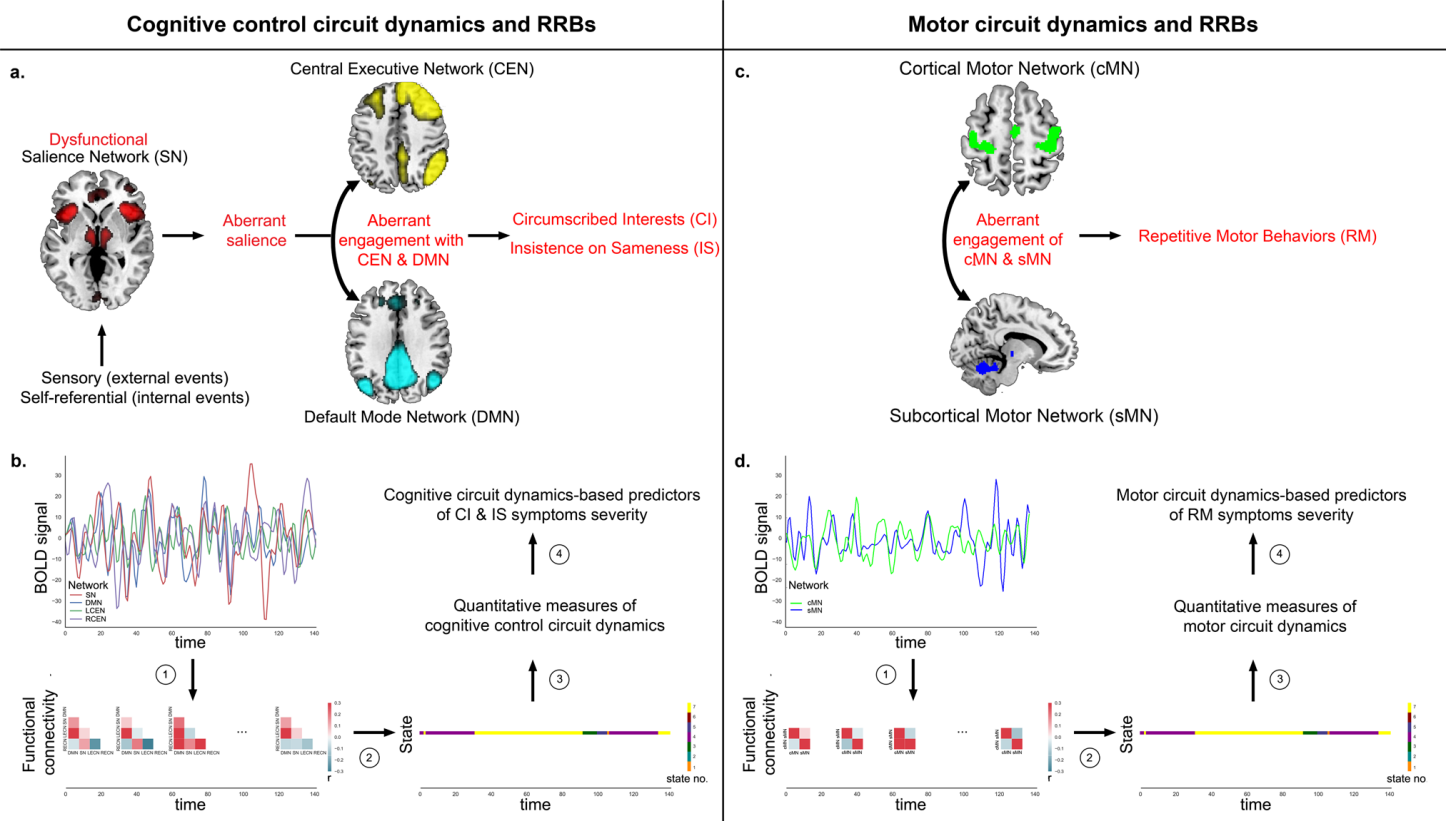
Overall approach to determine the temporal dynamics of cognitive control and motor circuits and its relationship with Restricted and Repetitive Behaviors (RRBs). **a** Cognitive control circuit-based model of circumscribed interests (CI) and insistence on sameness (IS) symptoms. The model proposes that aberrant functional of organization of key fronto-parietal-opercular cognitive control circuit may contribute to the cognitive components of RRBs i.e., CI and IS symptoms in children with ASD. Specifically, this model posits a key role for the salience network (SN) in aberrant mapping of internal and external salient events leading to altered dynamic temporal interactions with the central executive network (CEN), and the default mode network (DMN), resulting in CI and IS. **b** Overall analysis pipeline for examining dynamic time-varying cross-network interactions within cognitive control circuit and their relationship with CI and IS symptoms. Time-varying cross-network interaction was measured using a dynamic functional connectivity approach. (1) We estimated dynamic functional interactions between SN, CEN, and DMN using a sliding-window approach. (2) To identify distinct group-specific states associated with dynamic functional connectivity, we applied a group-wise k-means clustering on the time-series of correlation matrices in each group separately. (3) Brain state-specific network interaction index (NII) was used to characterize cross-network interaction in each dynamic brain state. NII for each state k by averaging NII across sliding-windows labeled as state k. Cognitive NII (CNII) of a sliding window was computed as the difference in correlation between SN and CEN time series and correlation between SN and DMN. The correlation values were extracted from the covariance matrix associated with that sliding window. Mean and variability of time-varying CNII was calculated as average and standard deviation of CNII values across dynamics brain states respectively. (4) Linear regression analysis was used to examine the relation between dynamic time-varying cross-network interactions measure, including mean and variability of time-varying NII, and ADI-R derived RRB subtype symptom severity scores. **c** Motor circuit-based model of repetitive motor behavior (RM) symptoms. The model proposes that aberrant functional of organization of key cortical-subcortical motor circuit may contribute to the motoric components of RRBs i.e., RM symptoms in children with ASD. Specifically, this model posits a key role for altered dynamic temporal interactions between the cortical motor network (cMN), and the subcortical motor network (sMN), resulting in RM. **d** Overall analysis pipeline for examining dynamic time-varying cross-network interactions within motor circuit and their relationship with RM symptoms.

Here we address critical gaps in our knowledge regarding heterogeneous expression of RRBs in childhood ASD using dynamic brain circuit analysis. We test the hypothesis that brain circuit dynamics underlying RRB symptoms can be dissociated, and specifically that, aberrant cognitive control circuit dynamics would underlie CI and IS symptoms whereas aberrant motor circuit dynamics would underlie RM symptoms. We characterize the dynamic properties of two distinct brain circuits: (i) a cognitive control circuit consisting of salience (SN)^16–18^, central executive (CEN)^18–20^, and default-mode (DMN)^21,22^ network nodes that play a key role in salience detection, allocation of attentional resources, and flexible behavior and (ii) a motor circuit, consisting of cortical (cMN) and subcortical (sMN) motor network nodes important for implementing motor planning, control, and execution^23^ (Fig. 1a). We predicted that compared to TD children, children with ASD would show less flexible, aberrant time-varying brain circuit dynamics. In addition, we predicted that aberrant dynamics of the cognitive control circuit would be associated with CI and IS symptoms of RRB, but not RM symptoms and that aberrant dynamics of the motor circuit would be associated with RM symptoms of RRB, but not CI and IS symptoms. Finally, we predicted that, compared to static functional circuits, dynamic functional circuits would better distinguish and predict distinct RRB clinical symptoms.

## Results

### RRB subtypes derived from the ADI-R

Because age and IQ can influence RRB factor structure^5^, we first examined a cohort (*N* = 126) of ASD participants in age and IQ matched to our imaging cohort. We performed principal component analysis (PCA) with varimax rotation on 9 Autism Diagnostic Interview-Revised (ADI-R) item-level scores^24,25^ (Supplementary Table 2). PCA found a three-component solution as the best solution, consistent with findings from previous studies albeit with different loadings^8,9^ (Supplementary Table 2). This three-component solution explained 54% of the variance, and each component included two to three items, as follows: (i) CI, which included, “Item 68: Circumscribed Interests” and “Item 76: Unusual Attachment to Objects”, (ii), IS, which included “Item 67: Unusual Preoccupations” and “Item 70: Compulsions and Rituals”, and (iii) RMB, which included “Item 69: Repetitive Use of Objects or Interests in Part of Objects”, “Item 77: Hand and Finger Mannerisms” and “Item 78: Other Complex Mannerisms or Stereotyped Body Movements” (Supplementary Table 2). CI, IS, and RM scores derived from our sample were strongly correlated with scores derived using factor weights from a previously published study by Lam and colleagues^9^ (Spearman *ρ*_CI_ = 0.88, *p* < 0.001, *ρ*_IS_ = 0.71, *p* < 0.001, *ρ*_RM_ = 0.86, *p* < 0.001) (see Supplementary Results for details).

### Temporal dynamics of cognitive control circuit in children with ASD and TD children

**Fig. 2:**
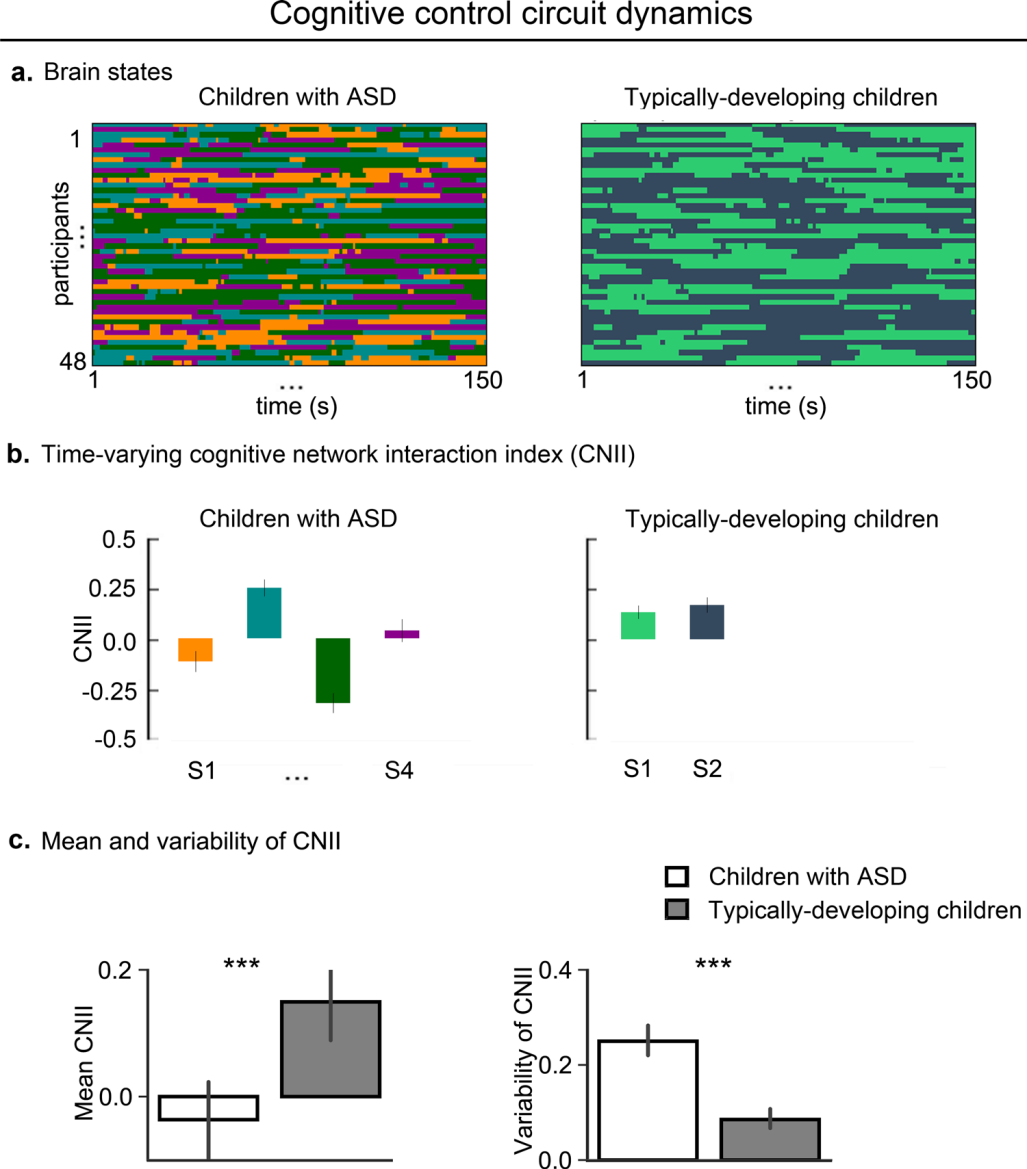
Aberrant temporal dynamics of cognitive control circuit in children with ASD. **a** Children with ASD showed four states and TD children showed two states. Color codes distinct states in each participant. States 1, 2, 3, and 4 in children with ASD are represented by dark orange, dark cyan, dark green, and dark magenta, respectively; states 1 and 2 in TD children are represented by lime green and dark blue, respectively. **b** Cognitive NII (CNII) of dynamic brain states showed intermittently reduced cross-network interaction in children with ASD compared to TD children. **c** The temporal mean of dynamic cross-network interaction in the cognitive control circuit, assessed using mean of dynamic CNIIs across states, was significantly lower in children with ASD, compared to TD children. The temporal variability of dynamic cross-network interaction in the cognitive control circuit, assessed using standard deviation of dynamic CNIIs across states, was significantly higher in children with ASD, compared to TD children. Two-sided two sample *t*-tests were used to compare mean and temporal variability of CNIIs between children with ASD and TD children. Error bar shows standard error of mean. ***p < 0.001.

We next examined dynamic, time-varying, cross-network functional interactions in the cognitive control circuit and found four states (temporal clusters) in children with ASD and two in TD children (Fig. 2a, b), reflecting variation in cross-network interactions across time in both groups. Importantly, these results argue against assumptions of stationarity made in most previous functional connectivity studies in ASD.

We then compared the cognitive network interaction index (CNII) of dynamic brain states between the two groups. We computed CNII for each sliding window and averaged CNII for the windows corresponding to the same dynamic brain state. The mean CNII value, averaged across all states, was significantly lower in the ASD group, compared to the TD group (*p* < 0.0001, *t*(94) = −4.07, Cohen’s *d* = 0.83) (Fig. 2c), even after controlling for confounds such as age, sex, head motion, and IQ (Supplementary Table 3). These results demonstrate an intermittent lack of integration of the SN with the CEN and reduced decoupling of the SN from the DMN in children with ASD.

We next compared variability of dynamic time-varying cross-network interactions between the two groups and found that compared to TD children, children with ASD showed greater variability in CNII values across states, suggesting that cross-network interactions in the cognitive control circuit are more variable in ASD than TD group (*p* < 0.0001, *t*(94) = 7.27, Cohen’s *d* = 1.48) (Fig. 2c), even after controlling for confounds (Supplementary Table 4).

The aforementioned results were also observed for a different sliding window length (=40 s) as well as for a different sliding window shape (rectangular), demonstrating that the findings are robust against the length and shape of the sliding window.

### Relation between temporal dynamics of cognitive control circuit and RRB subtypes in children with ASD

To investigate the extent to which atypical temporal dynamics of the cognitive control circuit is associated with severity of RRB subtypes in ASD, we examined the relationship between features of cognitive control circuit dynamics described above and ADI-R RRB factor scores.

Multivariate regression analysis revealed that mean and variability of CNII predicted CI scores (*F*(2, 45) = 3.9, *p* < 0.05) and IS scores (*F*(2, 45) = 3.3, *p* < 0.05) (see below). There was no significant relationship between mean and variability of CNII and RM (*F*(2, 45) = 0.48, *p* = 0.63) (Supplementary Fig. 4), emphasizing the specificity of the finding with CI and IS symptoms. To further examine the predictive ability of CNII, we performed a fivefold cross-validation analysis. Results from this analysis were consistent with the results from the original analysis (*r*(pred, actual)_CI_ = 0.29, *p*_CI_ = 0.01; *r*(pred, actual)_IS_ = 0.33, *p*_IS_ = 0.01; *r*(pred, actual)_RM_ = −0.08, *p*_RM_ = 0.40). Similar results were observed with a tenfold cross-validation analysis (see Supplementary Materials for details), highlighting the stability of the findings. This finding was replicated with CI, IS, and RM scores computed using RRB factor weights previously published by Lam and colleagues^9^ (see Supplementary Results). Importantly, none of the aforementioned brain-behavior relations were detected with measures of static/time-averaged functional interactions in the cognitive control circuit, demonstrating the specificity of the findings to dynamical properties of the cognitive control circuit (see Supplementary Materials for details).

### Temporal dynamics of motor circuit in children with ASD and TD children

**Fig. 3:**
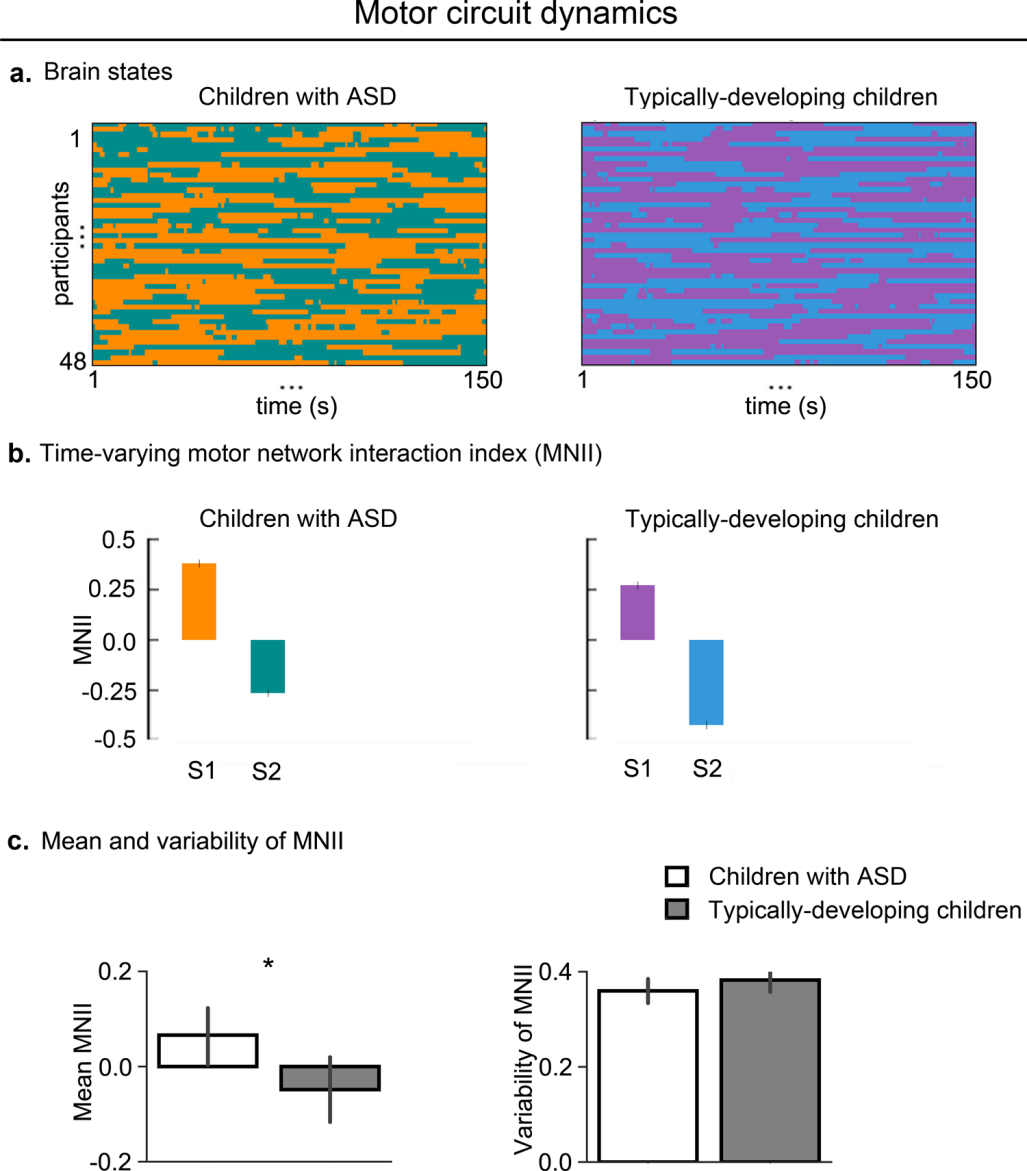
Aberrant temporal dynamics of motor circuit in children with ASD. **a** Children with ASD and TD children showed two states. Color codes distinct states in each participant. States 1 and 2 in children with ASD are represented by dark orange and dark cyan, respectively; states 1 and 2 in TD children are represented by moderate violet and bright blue, respectively. **b** Motor NII (MNII) of dynamic brain states showed intermittently increased cross-network interaction in children with ASD compared to TD children. **c** The temporal mean of dynamic cross-network interaction in the motor circuit, assessed using mean of dynamic MNIIs across states, was significantly higher in children with ASD, compared to TD children. The temporal variability of dynamic cross-network interaction in the motor circuit, assessed using standard deviation of dynamic MNIIs across states, did not differ between children with ASD and TD children. Two-sided two sample *t*-tests were used to compare mean and temporal variability of MNIIs between children with ASD and TD children. Error bar shows standard error of mean. *p < 0.05.

We next examined dynamic cross-network functional interactions in the motor circuit and found two states (temporal clusters) in children with ASD and two in TD children (Fig. 3a, b), reflecting variation in cross-network interactions across time in both groups, similar to the finding of cognitive control circuit.

We then compared the motor network interaction index (MNII) of dynamic brain states between the two groups. The mean MNII value was significantly higher in the ASD group, compared to the TD group (*p* < 0.05, *t*(94) = 2.3, Cohen’s *d* = 0.47) (Fig. 3c), even after controlling for confounds (Supplementary Table 5). These results demonstrate an intermittent increased coupling between the cMN and sMN in children with ASD.

We next compared variability of dynamic time-varying cross-network interactions between the two groups and found that variability in MNII values across states did not differ between the two groups (*p* = 0.23, *t*(94) = −1.2, Cohen’s *d* = 0.25) (Fig. 3c).

The aforementioned results were also observed for a different sliding window length (=40 s) as well as for a different sliding window shape (rectangular), demonstrating that the findings are robust against the length and shape of the sliding window.

### Relation between temporal dynamics of motor circuit and RRB subtypes in children with ASD

**Fig. 4:**
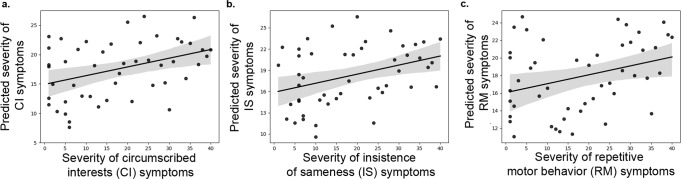
Aberrant brain circuit dynamics in children with ASD predict RRB subtypes severity. Regression analysis revealed that temporal mean and variability of dynamic cross-network interactions in the cognitive control circuit predicted **a** CI and **b** IS symptoms, but not RM symptoms. **c** Regression analysis revealed that temporal mean of dynamic cross-network interactions in the motor circuit predicted RM symptoms, but not CI or IS symptoms. Error band represent 95% confidence interval for the regression estimate. Cross-validation analyses confirmed these results.

To investigate the extent to which atypical temporal dynamics of the motor circuit is associated with severity of RRB subtypes in ASD, we examined the relationship between features of motor circuit dynamics described above and ADI-R RRB factor scores. Regression analysis revealed that mean of MNII predicted RM scores (*F*(1, 46) = 5.2, *p* < 0.05) (Fig. 4c). There was no significant relationship between mean MNII and CI (*F*(1, 46) = 0.45, *p* = 0.5) and IS (*F*(1, 46) = 3.3, *p* = 0.07) (Supplementary Fig. 4), emphasizing the specificity of this finding. To further examine the predictive ability of MNII, we performed fivefold cross-validation analysis. Results from this analysis were consistent with the results from the original analysis (*r*(pred, actual)_RM_ = 0.29, *p*_RM_ = 0.005; *r*(pred, actual)_CI_ = −0.20, *p*_CI_ = 0.53; *r*(pred, actual)_IS_ = 0.13, *p*_IS_ = 0.09). Similar results were observed with a tenfold cross-validation analysis (see Supplementary Materials for details), highlighting the stability of the findings. This finding was replicated with RM, CS, and IS scores computed using RRB factor weights previously published by Lam and colleagues^9^ (see Supplementary Results). Importantly, the brain-behavior relation was not detected with measures of static/time-averaged functional interactions in the motor circuit, demonstrating the specificity of the findings to dynamical properties of the motor circuit (see Supplementary Materials for details).

## Discussion

The present study addresses a critical gap in our knowledge of brain circuits underlying CI, IS, and RM, three distinct symptom clusters that define RRB—a core clinical phenotype of ASD—in affected children. Using a systems neuroscience-based approach and dynamic functional circuits analysis we provide evidence that childhood ASD is characterized by aberrant dynamics of multiple functional brain circuits, and crucially, that cognitive (CI, IS) and motoric (RM) RRB symptoms are associated with unique neurobiological signatures. Our findings demonstrate that dynamic properties of brain circuits can provide fundamental insights into mechanisms underlying heterogeneity of clinical symptoms in ASD.

Our dynamic connectivity analysis revealed that children with ASD have less flexible cognitive control circuit dynamics, characterized by brain states with impaired coupling of the SN with CEN and DMN, consistent with findings from a recent study that reported that adults with ASD show dominant neural states with aberrant functional interactions between SN and CEN and between SN and DMN^26^. Notably, we found that CI and IS symptoms of RRB were associated with the degree of inflexible interactions between the three key cognitive control networks: SN, CEN, and DMN^16,27^. Specifically, severity of CI and IS symptoms was associated with aberrant temporal engagement of the SN with the CEN and DMN. Notably, no such relation was found with RM symptoms pointing to the specificity of our findings with respect to cognitive inflexibility. Critically, no static time-averaged functional connectivity measures predicted CI or IS symptoms. These findings demonstrate that aberrant circuits dynamics associated with SN, CEN, and DMN carry clinically relevant neurobiological signatures of cognitive, but not motoric, components of RRB.

Cross-network interactions between the SN, CEN, and DMN play a key role in effectively responding to dynamic demands of changing environment^16,27^. In particular, interactions of the SN with the CEN and the DMN are thought to facilitate switching between externally-oriented attention and internally-oriented mental processes in response to salient events to guide flexible behavior^16,27^. Our dynamic network analysis revealed that this switching is impaired in children with ASD, and that the degree of impairments predicts cognitive inflexibility. These results are consistent with and extend previous studies based on static time-averaged measures demonstrating hyper-connectivity within the SN, CEN, and DMN in children with ASD^28^. Aberrant functioning of the anterior insula node of the SN in ASD^29^ may be a key mechanism contributing to inflexible circuits and behaviors given its key role as causal hub for switching between these networks^16,27^. Together, results suggest that reduced cross-network interactions in the cognitive control circuit contribute to core phenotypic features and inflexible behaviors such as intense focus, unusual attachment to objects of interest, and difficulty with changes in the environment, and provide support for a neurocognitive model of RRB^7^ in ASD based on dynamic circuit properties.

Analysis of motor circuit dynamics revealed a different pattern of association with specific RRB phenotypic features. Children with ASD had less flexible motor circuits characterized by stronger intermittent coupling between sMN and cMN. Moreover, we found a strong association between aberrant motor circuit dynamics and RM, but not CI and IS, pointing to the specificity of our findings with respect to motor symptoms. Our results highlight a tight link between sMN-cMN dynamics and RM symptoms observed in children with ASD.

The sMN and cMN nodes including the cerebellum, motor, and premotor regions are critical for motor control and execution^23^, and have been shown to have structural abnormalities in individuals with ASD^30,31^. Our results suggest reduced differentiation of these motor networks^32^ can lead to more rigidity in motor behaviors. We previously suggested that intrinsically hyper-connected circuits may make it more difficult to modulate connectivity in response to task demands, thereby resulting in task-related under-connectivity compared to the baseline state^33^. Consistent with this proposal, a previous study reported reduced static connectivity between the subcortical and cortical motor regions during a finger sequencing task in individuals with autism^34^. Thus, we hypothesize that the propensity of children with ASD to remain in brain states in which sMN and cMN nodes are intrinsically hyper-connected, potentially due to structural deficits in the fronto-thalamo-cerebellum pathway^32^, could lead to inflexible motor control^34,35^ and RM behaviors that are characteristic of the disorder^1^.

To address growing concerns about reproducibility of neuroscientific findings^36^, we leveraged our sample and conducted cross-validation analyses following procedures typically used in machine learning. Cross-validation is a powerful approach for validating research findings, and its use for demonstrating generalization and reproducibility has been advocated in psychiatry, psychology, and many other disciplines^37,38^. The results of these analyses were consistent with our original results, demonstrating the robustness of our findings. Finally, findings were replicated with RRB measures derived from a previously published factor structure^9^.

In conclusion, the present study is the first to demonstrate that CI, IS, and RM behaviors, the three phenotypic components of RRB, are associated with distinct features of brain circuit dynamics in childhood ASD. In contrast, such brain-clinical symptom associations were not observed with static time-averaged connectivity measures. Future work is needed to determine whether these findings, (i) observed in a predominantly male sample consistent with extant neuroimaging studies of ASD, extend to females with ASD, (ii) using 6 min fMRI scans, extend to longer scans, and (iii) using ADI-R, extend to other RRB scales such as Repetitive Behavioral Scale-Revised (RBS-R). Our findings of inflexible functional circuits provide a dynamic brain circuit model of RRB subtypes. Identification of unique neurobiological signatures underlying these symptoms may assist in the development of symptom-based biomarkers and treatments, including TMS that target cognitive control and motor circuits deficits identified here, in affected individuals. Importantly, our findings provide, to the best of our knowledge, novel neurobiological support for the validity of RRB subtypes. More generally, the parsimonious predictive framework and computational methods developed here may prove widely useful for better characterizing clinical heterogeneity in other psychiatric disorders based on systems-neuroscience based models of brain circuit dynamics.

## Methods

### Participants

The clinical part of the study wherein we examined the structure of the RRB symptoms included 126 children with ASD (112 males, 14 females; age: 10.0 ± 1.6 years; IQ: 110 ± 16). The imaging part of the study included: 48 children with ASD (41 males, 7 females; age: 10.9 ± 1.9 years; IQ: 115 ± 16) and 48 age- and gender-matched TD children (41 males, 7 females; age: 10.9 ± 1.7 years; IQ: 118 ± 11) (Supplementary Table 1 and Supplementary Fig. 1). Informed written consent was obtained from the legal guardian of each child and the study protocol was approved by the Stanford University Institutional Review Board. The ASD diagnosis procedure and the subject inclusion/exclusion criteria are described in detail in Supplementary Materials.

We also conducted a search (Supplementary Fig. 2) of publicly-available open-source ASD datasets including ABIDE (http://fcon_1000.projects.nitrc.org/indi/abide/) and the NIMH Data Archive (https://nda.nih.gov/) and found that none of these data contain item-level ADI-R^24,25^ scores, crucial phenotypic data relevant to our study, highlighting the uniqueness of our data.

### Clinical measures and analysis

Similar to previous studies, we used ADI-R to assess RRBs in each child with ASD^8,9^. To determine the factor structure of the RRB symptom domain as measured by the ADI-R, we performed a PCA with varimax rotation on ADI-R items, using Matlab R2018v5.0. In line with procedures described in previous studies^8,9^, nine ADI-R items which assess RRBs were included in the analysis (Supplementary Table 2). “Current” behavior ratings were used and scores of 6, 7, and 8 were converted to 0. The number of components extracted in PCA was determined using a combination of eigenvalues above 1 and scree plot.

### Imaging data acquisition

Each participant underwent a 6-min resting-state fMRI scan and a T1-weighted structural imaging scan on a 3 T GE Signa scanner in the same session. Participants were instructed to stay awake, keep their eyes closed, and try not to move for the duration of the 6-min scan. Imaging data acquisition protocol and parameters are described in detail in Supplementary Materials.

### Imaging data analysis

Overall imaging data analysis pipeline is illustrated in Fig. 1. Imaging data were preprocessed^14^ (see Supplementary Materials for details) and the preprocessed resting-state fMRI data were entered into a group independent component analysis (ICA) to identify SN, left CEN, right CEN, DMN, cMN, and sMN.

#### Dynamic functional brain circuit analysis

#### Cognitive control circuit

We applied dynamic functional connectivity analysis on the SN, CEN, and DMN timeseries data in each group (see Supplementary Materials for details and Supplementary Fig. 3). Briefly, we first estimated dynamic functional interactions between SN, CEN, and DMN using an exponentially decaying sliding window. Second, we identified distinct group-specific states associated with dynamic functional connectivity, using group-wise k-means clustering. The optimal number of clusters, i.e., states, was determined using maximal silhouette across multiple iterations^39^. Because our goal was to investigate whether dynamic temporal properties differed between the two groups (children with ASD and TD children), we allowed the number of clusters to differ between the groups, instead of keeping them exactly the same^14^. Third, we characterized cross-network interaction in each dynamic brain state, using brain state-specific CNII. CNII measures cross-network interactions among the three networks based on the hypothesized role of the SN in switching interactions with the CEN and DMN^16,17^. CNII has the advantage of capturing interactions simultaneously among all three networks. Specifically, CNII was computed as the difference in correlation between SN and CEN time series and correlation between SN and DMN. CNII thus captures the extent to which SN temporally engages with CEN and dissociates itself from DMN^21,17^. We computed CNII for each sliding window and the (i) mean and (ii) variability (measured by standard deviation) of time-varying CNII across all the dynamic brain states for each participant. We then examined the difference between the mean and variability of time-varying CNII between the two groups using two sample *t*-tests. Non-parametric linear regression was used to test associations between time-varying functional connectivity metrics of the cognitive control circuit and RRB subtypes. To further examine the predictive ability of cognitive control circuit dynamics and assess reproducibility, we leveraged our sample and conducted fivefold and tenfold cross-validation analyses^37,38,40^ (see Supplementary Materials for details).

#### Motor circuit

We applied dynamic functional connectivity analysis procedures on the cMN and sMN timeseries data in each group (see Supplementary Materials for details and Supplementary Fig. 3). Briefly, we first estimated dynamic functional interactions between cMN and sMN using an exponentially decaying sliding window. Second, we identified distinct group-specific states associated with dynamic functional connectivity, using group-wise k-means clustering. Third, we characterized cross-network interaction in each dynamic brain state, using brain state-specific MNII. MNII measures cross-network interactions among the two networks involved in motor function and was computed as the correlation between cMN and sMN time series. MNII thus captures the extent to which cMN temporally engages with sMN. We computed MNII for each sliding window and the (i) mean and (ii) variability (measured by standard deviations) of time-varying MNII across all the sliding windows for each participant. We then examined the difference between the mean and variability of time-varying MNII between the two groups using two sample *t*-tests. Non-parametric linear regression was used to test associations between time-varying functional connectivity metrics of the motor circuit and RRB subtypes. To further examine the predictive ability of motor circuit dynamics, we used fivefold and tenfold cross-validation analyses (see Supplementary Materials for details).

### Reporting summary

Further information on research design is available in the Nature Research Reporting Summary linked to this article.

## Supplementary information

Supplementary Information

Reporting Summary

## Data Availability

All data that support the findings of this study are available from the corresponding authors upon reasonable request.

## Code availability

Data were analyzed using Matlab 9.5 (R2018b) and SPM8. Data analysis scripts will be made available via GitHub upon publication.
