# Supplementary material for: Aberrant dynamics of cognitive control and motor circuits predict distinct restricted and repetitive behaviors in children with autism
Source: Nat Commun. 2021 Jun 10;12:3537. doi: 10.1038/s41467-021-23822-5 (PMC8192778; doi:10.1038/s41467-021-23822-5)
Supplement: Supplementary file 1 — Supplementary Information [file 41467_2021_23822_MOESM1_ESM.pdf]

Supplementary Materials

I. Supplementary Methods

Participants

ADI-R Factor Analysis. One hundred and twenty-six children with ASD (112 males, 14 females; age: 10.0 ± 1.6 years; IQ: 110 ± 16) participated in this study after written informed consent was obtained from their legal guardian. The study protocol was approved by the Stanford University Institutional Review Board. Participants were recruited locally, from schools and clinics near Stanford University. All children were required to have a Full Scale IQ > 70, as measured by the Wechsler Abbreviated Scale of Intelligence (WASI[1]).

fMRI. Forty-eight children with ASD (41 males, 7 females; age: 10.9 ± 1.9 years; IQ: 115 ± 16) and 48 age- and gender-matched TD children (41 males, 7 females; age: 10.9 ± 1.7 years; IQ: 118 ± 11) participated in this study after written informed consent was obtained from their legal guardian (Supplementary Table 1, Supplementary Figure 1). The study protocol was approved by the Stanford University Institutional Review Board. Participants were recruited locally, from schools and clinics near Stanford University. All children were required to have a Full Scale IQ > 70, as measured by WASI.

Children with ASD received a diagnosis based on scores from the ADI-R [2,3] and/or the Autism Diagnostic Observation Schedule (ADOS) [4] following criteria established by the National Institute of Child Health & Human Development/National Institute of Deafness and Other Communication Disorders Collaborative Programs for Excellence in Autism [5]. Children with

ASD were screened through a parent phone interview and excluded if they had any history of known genetic, psychiatric, or neurological disorders (e.g., Fragile X syndrome or Tourette's syndrome), or were currently prescribed anti-psychotic medications. TD children were screened and excluded if they or a first-degree relative had developmental, language, learning, neurological, psychiatric disorders, or psychiatric medication usage, or if the child met the clinical criteria for a childhood disorder on the Child Symptom Inventory – Fourth Edition or Child and Adolescent Symptom Inventory. All participants underwent a battery of standardized neuropsychological assessments including WASI [1], and the Wechsler Individual Achievement Test (WIAT, 2nd edition).

ADI-R factor analysis

To determine RRB subtypes, we applied principal component analysis (PCA) with varimax rotation on 9 ADI-R items that assess RRBs[6] (Supplementary Table 2). The number of factors was determined by a combination of scree plot and eigenvalue greater than 1[6].

fMRI data acquisition

For each subject a resting-state fMRI scan was acquired using the following protocol. Functional images were acquired on a 3T General Electric (GE) Signa scanner using a custom-built head coil. Head movement was minimized during scanning by small foam cushions placed on the sides of the subject's head. A total of 29 axial slices (4.0 mm thickness, 0.5 mm skip) parallel to the AC-PC line and covering the whole brain were imaged with a temporal resolution of 2 s using a T2* weighted gradient echo spiral in-out pulse sequence [7] with the following parameters:

TR = 2,000 msec, TE = 30 msec, flip angle = 80 degrees, 1 interleave. The field of view was 20 cm, and the matrix size was 64×64, providing an in-plane spatial resolution of 3.125 mm. To reduce blurring and signal loss arising from field in homogeneities, an automated high-order shimming method based on spiral acquisitions was used before acquiring functional MRI scans. Participants were repeatedly instructed to stay awake, keep their eyes closed and try not to move for the duration of the 6-min scan. To avoid the influence of task and prevent drowsiness, the resting-state scans were placed at the beginning of the scanning session. A T1-weighted structural imaging scan was also acquired in the same session.

fMRI data preprocessing and analysis

Preprocessing

A standard preprocessing procedure was implemented using SPM8, including slice-timing correction, realignment, normalization, spatial smoothing (6-mm smoothing kernel), regression of nuisance variables (24 motion parameters, signals from the white matter and CSF), and bandpass filtering (0.008 Hz < f < 0.1 Hz). The 24 motion parameters include [R R^2 R_{t-1} R_{t-1}^2], where t and t-1 refer to the current and immediately preceding timepoint and R = [X Y Z pitch yaw roll]. The motion parameters did not differ between the ASD and TD groups (Supplementary Table 1).

Network identification

Preprocessed fMRI data were entered into a group independent component analysis (ICA) to

identify large-scale networks in the combined population (MELODIC, http://fsl.fmrib.ox.ac.uk/fsl/fslwiki/MELODIC). The number of components was set to 30. Determining the number of components using an unsupervised learning algorithm like ICA remains an unresolved challenge. Our choice of 30 components was based on findings from a seminal study [8] that comprehensively evaluated the influence of the number of components on the accuracy of ICA results. The study found that the quality of ICA estimation does not improve once the ICA components are being estimated in a subspace with more than 30 dimensions and that reducing the number of components below 30 results in poor estimation. Four components (SN, left and right CEN, and DMN) corresponding to the previously described triple-network model of cognitive control [9] and two components (cMN, sMN) corresponding to the motor circuit were determined based on a widely-used visual inspection procedure [10,11]. Briefly, an expert (K.S.) examined the spatial and temporal profile of each of the 30 ICA components and labelled it as SN, DMN, Left CEN, Right CEN, cMN, sMN or other. These labels were further confirmed by a quantitative template-matching procedure [12]. The template matching procedure involved taking the average z score of voxels falling within the template minus the average z score of voxels outside the template and selecting the component in which this difference (the goodness of fit) was the greatest. The templates for SN, DMN, Left CEN, Right CEN, cMN and sMN were identified from previously published adult studies [13,14]. The template matching procedure is identical to those used in previous published studies [11].

Dynamic functional brain circuit analysis

Cognitive Control Circuit. Time-varying cross-network interaction was measured using a dynamic functional connectivity approach [15-17]. Our overall analysis pipeline is illustrated in

Figure 1B. We estimated dynamic functional interactions between SN, CEN, and DMN using an exponentially decaying sliding window and a window length of 50 seconds (25 TRs) and a sliding step of 2 seconds (1 TR) [15,18,19]. Exponentially decaying weights were applied to each time point within a window as described in previous studies [15,16]. Within each time window, we computed the z-transformed Pearson correlation between the ICA time-series taken pairwise. This resulted in a time-series of correlation matrices (T x C); here T is the number of time windows and C is number of pairwise interactions among SN, CEN, DMN at each time point. To identify distinct group-specific states associated with dynamic functional connectivity, we applied a group-wise k-means clustering on the time-series of correlation matrices in each group separately with the number of clusters (k) ranging from 2 to 20, using Matlab kmeans function. Twenty-five different initializations were used to reduce the chance of local minima. The number of initializations we used is considerably higher than the Matlab as well as Python sklearn recommended/default number of replicates (=10), while at the same time within reasonable computational capacity. Clustering performance was estimated using the silhouette method and the optimal number of clusters was determined based on maximal silhouette across all the iterations [20]. Because our goal was to investigate whether dynamic temporal properties differed between the two groups we allowed the number of clusters to differ between the children with ASD and TD children groups, instead of keeping them exactly the same [18]. Robustness of our findings was tested using different window lengths.

Brain state-specific cognitive network interaction index (CNII) was used to characterize cross-network interaction in each dynamic brain state. CNII measures cross-network interactions among the three networks based on the hypothesized role of the SN in switching interactions

with the CEN and DMN [9,21]. CNII has the advantage of capturing interactions simultaneously among all three networks. Specifically, CNII was computed as the difference in correlation between SN and CEN time series and correlation between SN and DMN. The rationale here is that SN and CEN are typically co-activated during cognitively demanding tasks, while SN and DMN are typically anti-correlated [21,22]. CNII thus captures the extent to which SN temporally engages with CEN and dissociate itself from DMN.

$$CNII = f(CC_{SN,CEN}) - f(CC_{SN,DMN})$$

where

$$f(CC) = \frac{1}{2}\ln\left(\frac{1+CC}{1-CC}\right)$$

CC is Pearson's correlation between the time series of two component networks, e.g., $CC_{\mathrm{SN,\ DMN}}$ refers to correlation between the time series of SN and DMN. $f(CC)$ computes Fisher z-transform of Pearson Correlation (CC) between ROI timeseries. Thus for instance, $f(CC_{SN,CEN})$ computes Fisher z-transform of Pearson Correlation between the time series of SN and CEN. $f(CC_{SN,LCEN})$ and $f(CC_{SN,RCEN})$ were computed separately and then their average was used as $f(CC_{SN,CEN})$. Larger CNII values reflect more segregated cross-network interactions between the SN-CEN and SN-DMN systems in the context of the triple-network model. We computed CNII for each sliding window, and then computed the (i) mean and (ii) variability (measured by standard deviations) of time-varying CNII across all the dynamic brain states for each participant and examined the difference between the mean and variability of time-varying CNII between the two groups using two sample *t*-tests.

Motor Circuit. Time-varying cross-network interaction was measured using a similar dynamic functional connectivity approach [15-17]. Our overall analysis pipeline is illustrated in Figure 1D. Briefly, we first estimated dynamic functional interactions between cMN and sMN using an exponentially decaying sliding window. Second, we identified distinct group-specific states associated with dynamic functional connectivity, using the group-wise 1D k-means clustering. Third, we characterized cross-network interaction in each dynamic brain state, using brain state-specific motor network interaction index (MNII). MNII measures cross-network interactions among the two networks involved in motor function and was computed as the correlation between cMN and sMN time series. MNII thus captures the extent to which cMN temporally engages with sMN. We computed MNII for each sliding window, and then computed the (i) mean and (ii) variability (measured by standard deviations) of time-varying MNII for each participant and examined the difference between the mean and variability of time-varying MNII between the two groups using two sample *t*-tests.

Prediction analysis to determine the relation between temporal dynamics of cognitive control circuit and RRB subtypes in children with ASD

We used regression analysis to examine the relation between temporal dynamics of cognitive control circuit and RRB subtypes in children with ASD. Mean and variability of CNII as independent variables and RRB subtype (CI, IS or RM) severity score as dependent variable was used as the input to a non-parametric linear regression algorithm. Both mean and variability of CNII were used as independent variables as they were significantly different between the children with ASD and TD children groups. To further examine the predictive ability of cognitive control dynamics, we leveraged our sample and conducted cross-validation analyses

following procedures typically used in machine learning. Cross-validation is a powerful approach for validating research findings, and its use for demonstrating generalization and reproducibility has been advocated in psychiatry, psychology and many other disciplines[23,24]. Data were divided into five folds, consistent with the number of folds recommended for predictions studies[25]. A non-parametric linear regression model was built/trained using four folds, leaving out one fold. The samples in the left-out fold were then predicted using this trained model, and the predicted values were noted. This procedure was repeated five times, and finally an *r*(pred, actual) was computed based on the predicted and actual values. *r*(pred, actual), correlation between the predicted value of the trained linear regression model and the actual value, was used as a measure of how well the independent variable predicts dependent variable, with *r*(pred, actual) = 1 being the most accurate prediction model. Finally, the statistical significance of the model was assessed using nonparametric analysis. The empirical null distribution of *r*(pred, actual) was estimated by generating 1000 surrogate datasets under the null hypothesis that there was no association between temporal dynamics of cognitive control circuit and RRB subtype severity. Each surrogate dataset D_i of size equal to the observed dataset was generated by permuting the labels (dependent variable) on the observed data points. $r(\text{pred, actual})_i$ was computed using the actual labels of D_i and predicted labels using the five-fold cross-validation procedure described previously. This procedure produces a null distribution of *r*(pred, actual) for the regression model. The statistical significance (*p* value) of the model was then determined by counting the number of $r(\text{pred, actual})_i$ greater than *r*(pred, actual) and then dividing that count by the number of D_i datasets (1000 in our case). This analysis was conducted for three RRB subtypes, including CI, IS and RM. To assess the robustness of our approach we also repeated the aforementioned analysis with number of folds = 10.

Prediction analysis to determine the relation between temporal dynamics of motor circuit and RRB subtypes in children with ASD

We used regression analysis to examine the relation between temporal dynamics of motor circuit and RRB subtypes in children with ASD. Mean of MNII as independent variables and RRB subtype (CI, IS or RM) severity score as dependent variable was used as the input to a non-parametric linear regression algorithm. Mean of MNII was used as independent variable as it was significantly different between the children with ASD and TD children groups. To further examine the predictive ability of motor circuit dynamics, we used the five cross-validation approach described above. Data were divided into five folds. A non-parametric linear regression model was built/trained using four folds, leaving out one fold. The samples in the left-out fold were then predicted using this trained model, and the predicted values were noted. This procedure was repeated five times, and finally an r(pred, actual) was computed based on the predicted and actual values. r(pred, actual), correlation between the predicted value of the trained linear regression model and the actual value, was used as a measure of how well the independent variable predicts dependent variable, with r(pred, actual) = 1 being the most accurate prediction model. Finally, the statistical significance of the model was assessed using nonparametric analysis. The empirical null distribution of r(pred, actual) was estimated by generating 1000 surrogate datasets under the null hypothesis that there was no association between temporal dynamics of motor circuit and RRB subtype severity. Each surrogate dataset D_i of size equal to the observed dataset was generated by permuting the labels (dependent variable) on the observed data points. $r(\text{pred, actual})_i$ was computed using the actual labels of D_i and predicted labels using

the five-fold cross-validation procedure described previously. This procedure produces a null distribution of r(pred, actual) for the regression model. The statistical significance (p value) of the model was then determined by counting the number of $r(\text{pred, actual})_i$ greater than r(pred, actual) and then dividing that count by the number of D_i datasets (1000 in our case). This analysis was conducted for three RRB subtypes, including CI, IS and RM. To assess the robustness of our approach we also repeated the aforementioned analysis with number of folds = 10.

Open-source publicly-available data

We launched a search (Supplementary Figure 1) of publicly-available open source datasets. Specifically, we first examined in detail resting state fMRI and phenotypic data made available through the ABIDE I and ABIDE II initiatives (http://fcon_1000.projects.nitrc.org/indi/abide/). Although we were able to identify over 400 children with ASD and 400 TD children with good resting state fMRI data, none of the children with ASD had item-level ADI-R scores that are essential to determine RRB subtype severity scores. To address this, we requested item-level ADI-R scores from PIs of the ABIDE sites that included ASD children with good resting state fMRI data. Commendably, the PIs were quite prompt in their response, but unfortunately they did not have the resources to e-transcribe and share the item-level scores, which are collected in a paper form.

Our next quest for data led us to the National Institute of Mental Health Data Archive (NDA;https://nda.nih.gov/) – another open source dataset that makes available phenotypic and fMRI data from individuals with ASD and neurotypical individuals. Unfortunately, a

comprehensive examination of the NDA data yielded no participants who had both resting state fMRI data and item-level ADI-R scores. This exercise further highlights the uniqueness of our data and ensuing findings.

II. Supplementary Results

Ruling out potential confounds on between-group comparisons

Mean of dynamic time-varying CNII values were significantly different between children with ASD and TD children groups ($p < 0.05$, Supplementary Table 3), even after controlling for the potential confounding effects of age, movement, sex, and IQ.

Variability of dynamic time-varying CNII values were significantly different between children with ASD and TD children groups ($p < 0.05$, Supplementary Table 4), even after controlling for the potential confounding effects of age, movement, sex, and IQ.

Mean of dynamic time-varying MNII values were significantly different between children with ASD and TD children groups ($p < 0.05$, Supplementary Table 5), even after controlling for the potential confounding effects of age, movement, sex, and IQ.

Analysis of brain states

We compared mean dwell times across states and found that none of the states had mean dwell time significantly higher than other states, and none of the states had mean dwell time significantly lower than other states. This result was observed for brain states associated with cognitive control circuit dynamics as well as motor circuit dynamics in the ASD and TD groups suggesting that these brain states are equally probable.

Prediction analysis results with 10-fold cross validation.

Results of regression analysis with 10-fold cross validation were consistent with the results obtained with 5-fold cross validation, namely: (i) mean and variability of CNII was predictive of CI scores and IS scores, but not RM scores (r(pred, actual)$_{CI}$=0.29, p_{CI}=0.01; r(pred, actual)$_{IS}$=0.26, p_{IS}=0.01; r(pred, actual)$_{RM}$=-0.11, p_{RM}=0.42), (i) mean of MNII was predictive of RM scores, but not CI and IS scores (r(pred, actual)$_{RM}$=0.22, p_{RM}=0.02; r(pred, actual)$_{CI}$=-0.15, p_{CI}=0.38; r(pred, actual)$_{IS}$=0.14, p_{IS}=0.08).

Relationship between time-averaged cross-network functional interactions and RRB subtypes in children with ASD

We examined the relationship between time-averaged functional interactions in the cognitive control circuit and ADI-R RRB factor scores. None of the time-averaged cross-network interactions in the cognitive control circuit were associated with CI, IS and RM scores (all *p*'s > 0.05).

We examined the relationship between time-averaged functional interactions in the motor circuit and ADI-R RRB factor scores. Time-averaged cross-network interactions in the motor circuit were not significantly associated with CI, IS and RM scores (all *p's* > 0.05).

Robustness of brain-behavior findings against ADI-R factor structure

First, we repeated the PCA-based factor analysis using python as well as SPSS. The results were identical to those originally reported (which were obtained using Matlab code), confirming the accuracy of our procedures. Second, we examined the relation between our ADI-R PCA factor

weights/loadings (Supplementary Table 2) and those published previously by Lam and Colleagues[6] on our findings. We computed subject-wise CI, IS and RM scores using the previously published weights. We found a high correlation between CI, IS and RM scores computed using our weights and CI, IS and RM scores using the previously published weights (Spearman $\rho_{CI} = 0.88$, $p < 0.001$, $\rho_{IS} = 0.71$, $p < 0.001$, $\rho_{RM} = 0.86$, $p < 0.001$). Third, we examined the relationship between features of cognitive control circuit dynamics and CI, IS and RM scores that were computed using the previously published weights by Lam and colleagues. Cross-validation analysis revealed findings consistent with the results from the original analysis, namely: mean and variability of cognitive control circuit dynamics measure CNII was predictive of CI scores and IS scores, but not RM scores ($r(\text{pred, actual})_{CI}=0.47$, $p_{CI}=0.001$; $r(\text{pred, actual})_{IS}=0.22$, $p_{IS}=0.03$; $r(\text{pred, actual})_{RM}=-0.37$, $p_{RM}=0.86$). Fourth, we examined the relationship between features of motor circuit dynamics and CI, IS and RM scores that were computed using the previously published weights. Results from this cross-validation analysis were consistent with the results from the original analysis, namely: mean of motor circuit dynamics measure MNII was predictive of RM scores, but not CI and IS scores ($r(\text{pred, actual})_{RM}=0.34$, $p_{RM}=0.005$;$r(\text{pred, actual})_{CI}=0.02$, $p_{CI}=0.18$; $r(\text{pred, actual})_{IS}=0.11$, $p_{IS}=0.07$). Fifth, to further demonstrate the robustness of our findings, we examined CI, IS and RM scores by zeroing out the weights of the two items “resistance to trivial changes in the environment” and “difficulties in minor changes in subject’s environment” in our weights. Results from brain-behavior analyses using these CI, IS and RM scores were consistent with the results from the original analysis. Taken together, these results, further demonstrate the robustness of our main findings

Supplementary Tables

Supplementary Table 1. Descriptive statistics for the children with autism spectrum disorder (ASD) and typically-developing (TD) children groups. The two groups were matched on age, sex, intelligent quotient (IQ) and head motion during functional MRI. Two sided two-sample t-tests were used to compare age, IQ and head motion parameters between the children with ASD and TD groups. Two sided Chi-Squared test was used to compare sex distribution between the children with ASD and TD groups.

	**ASD (n = 48)**	**TD (n = 48)**	***p***
**Age**	10.9 ± 1.9 years	10.9 ± 1.7 years	0.99
**Sex (male/female)**	41/7	41/7	1
**IQ**	115 ± 16	118 ± 11	0.27
**Head Motion**			
Range			
X (mm)	0.57 ± 0.62	0.56 ± 0.56	0.97
Y (mm)	0.79 ± 0.61	0.74 ± 0.64	0.66
Z (mm)	1.53 ± 0.99	1.46 ± 1.06	0.72
Pitch (mm)	1.12 ± 1.06	0.97 ± 0.77	0.44
Roll (mm)	1.46 ± 1.24	1.43 ± 1.26	0.89
Yaw (mm)	0.66 ± 0.67	0.60 ± 0.64	0.64

Scan to Scan motion (mm)	0.15 ± 0.11	0.13 ± 0.06	0.17

Supplementary Table 2. Restricted and Repetitive Behavior (RRB) subtypes based on factor analysis of items from the Autism Diagnostic Interview-Revised (ADI-R).

ADI-R RRB Items	CI	IS	RM
68 Circumscribed Interests	.63	.06	.13
76 Unusual attachment to objects	.71	-.09	.01
75 Resistance to trivial changes in the environment	.73	.15	-.04
70 Compulsions/Rituals	.27	.69	.20
67 Unusual preoccupations	-.10	.83	.01
74 Difficulties in minor changes in subject's environment	.53	.07	.35
69 Repetitive use of objects or interest in parts of objects	.26	.34	.61
77 Hand and finger mannerisms	-.16	.22	.69
78 Other complex mannerisms/stereotyped body movements	.22	-.18	.71

Supplementary Table 3. Multiple linear regression revealed that, after controlling for all potential confounds, mean of dynamic time-varying cognitive network interaction index (CNII) was still significantly different between the children with autism spectrum disorder (ASD) and typically-developing (TD) children groups.

	t	*p*
Group	-4.16	**.00001**
Age	-.44	.66
Scan-to-Scan Motion	-1.67	.10
Sex	.05	.60
IQ	-.11	.31

Supplementary Table 4. Multiple linear regression revealed that, after controlling for all potential confounds, variability of dynamic time-varying cognitive network interaction index (CNII) was still significantly different between the children with autism spectrum disorder (ASD) and typically-developing (TD) children groups.

.

	t	*p*
Group	5.8	**.00001**
Age	-.40	.69
Scan-to-Scan Motion	1.29	.20
Sex	-.85	.40
IQ	-.79	.43

Supplementary Table 5. Multiple linear regression revealed that, after controlling for all potential confounds, mean of dynamic time-varying motor network interaction index (MNII) was still significantly different between the children with autism spectrum disorder (ASD) and typically-developing (TD) children groups.

	t	*p*
Group	2.57	**.012**
Age	-1.84	.07
Scan-to-Scan Motion	-.76	.45
Sex	-.71	.48
IQ	.59	.55

Supplementary Figures

Supplementary Figure 1. Subject selection procedure.

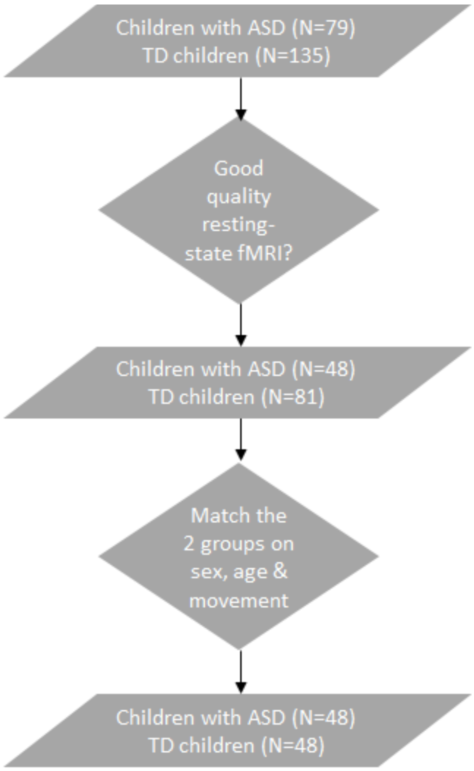

Supplementary Figure 2. Search of open-source autism spectrum disorder (ASD) datasets yielded no subjects who had both resting state functional MRI (fMRI) data and item-level Autism Diagnostic Interview-Revised (ADI-R) scores.

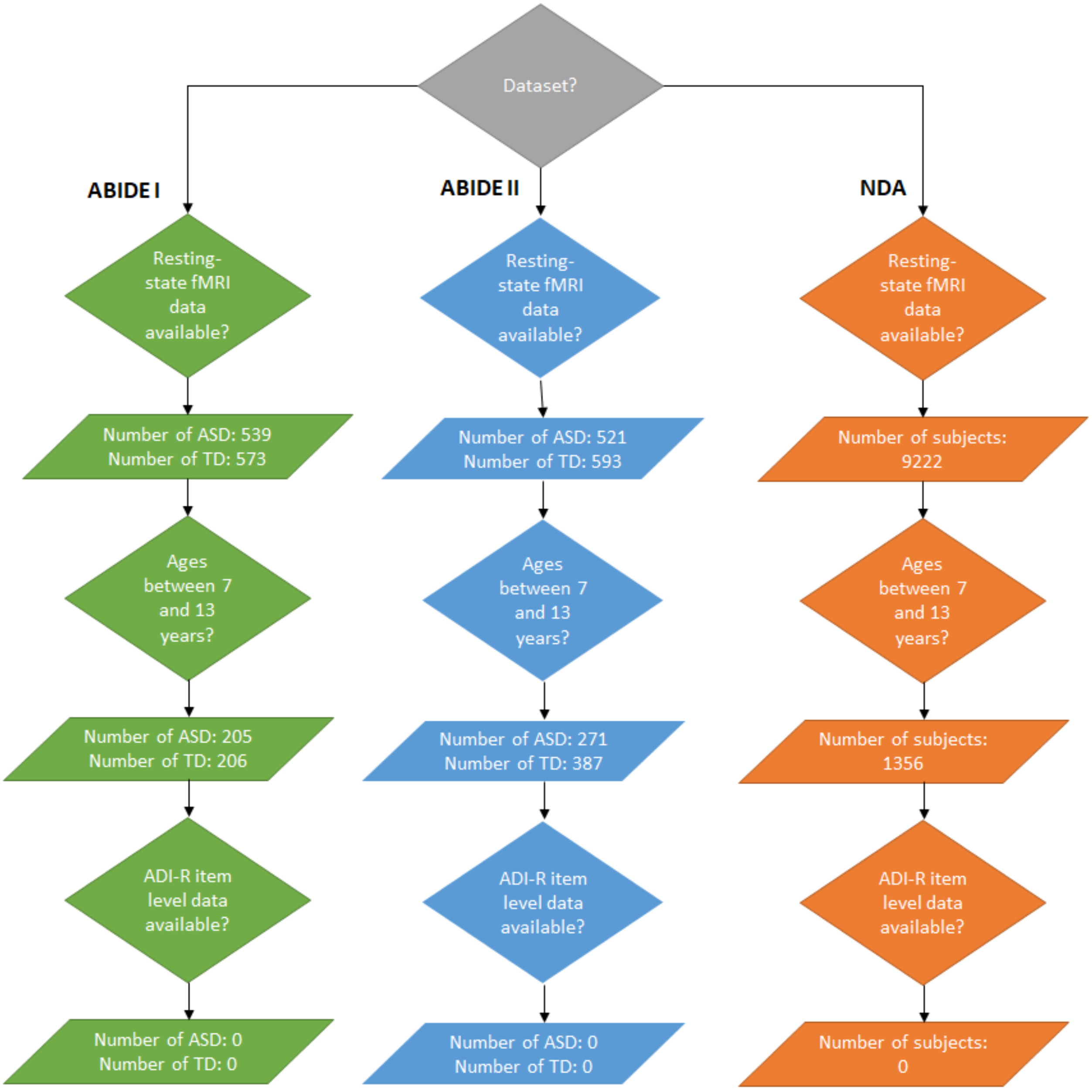

Supplementary Figure 3. Salience, Central Executive, Default mode, Cortical Motor and Subcortical Motor networks. (**A**) Salience Network (SN), (**B**) Left Central Executive Network (LCEN), (**C**) Right Central Executive Network (RCEN), (**D**) Default Mode Network (DMN), (**E**) Cortical Motor Network (cMN) and (**F**) Subcortical Motor Network (sMN).

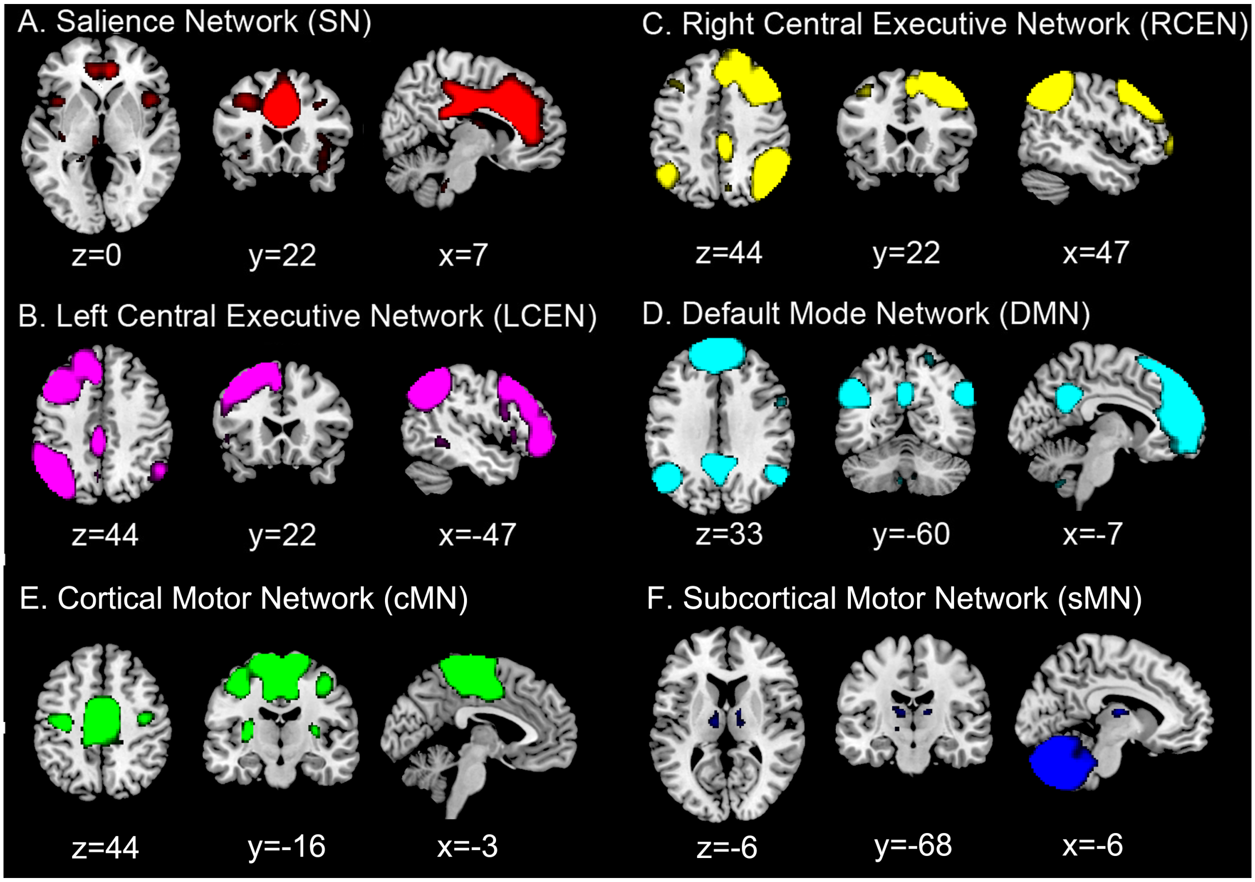

Supplementary Figure 4. Regression analysis revealed that temporal mean of dynamic cross-network interactions in the motor circuit do not predict **(a)** CI or **(b)** IS symptoms. **(c)** Regression analysis revealed that temporal mean and variability of dynamic cross-network interactions in the cognitive control circuit do not predict RM symptoms. Error band represent 95% confidence interval for the regression estimate. Cross-validation analyses confirmed these results.

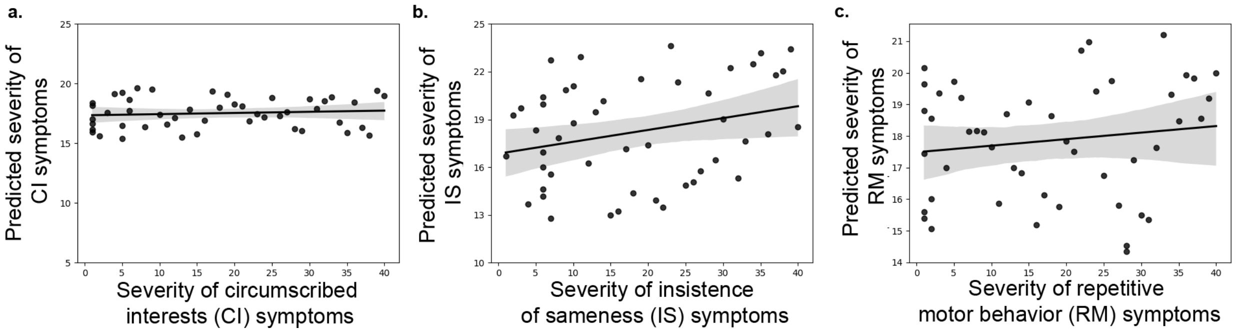

References

1 The Psychological, C. *Wechsler Abbreviated Scale of Intelligence*. (Harcourt Brace & Co., 1999).

2 Le Couteur, A. *et al.* Autism diagnostic interview: a standardized investigator-based instrument. *J Autism Dev Disord* **19**, 363-387 (1989).

3 Lord, C., Rutter, M. & Le Couteur, A. Autism Diagnostic Interview-Revised: a revised version of a diagnostic interview for caregivers of individuals with possible pervasive developmental disorders. *J Autism Dev Disord* **24**, 659-685 (1994).

4 Lord, C. Commentary: achievements and future directions for intervention research in communication and autism spectrum disorders. *J Autism Dev Disord* **30**, 393-398 (2000).

5 Lainhart, J. E. Advances in autism neuroimaging research for the clinician and geneticist. *Am J Med Genet C Semin Med Genet* **142C**, 33-39, doi:10.1002/ajmg.c.30080 (2006).

6 Lam, K. S., Bodfish, J. W. & Piven, J. Evidence for three subtypes of repetitive behavior in autism that differ in familiality and association with other symptoms. *J Child Psychol Psychiatry* **49**, 1193-1200, doi:10.1111/j.1469-7610.2008.01944.x (2008).

7 Glover, G. H. & Law, C. S. Spiral-in/out BOLD fMRI for increased SNR and reduced susceptibility artifacts. *Magnetic Resonance in Medicine* **46**, 515-522 (2001).

8 Beckmann, C. F. & Smith, S. M. Probabilistic independent component analysis for functional magnetic resonance imaging. *IEEE Trans Med Imaging* **23**, 137-152, doi:10.1109/TMI.2003.822821 (2004).

9 Menon, V. & Uddin, L. Q. Saliency, switching, attention and control: a network model of insula function. *Brain Struct Funct* **214**, 655-667, doi:10.1007/s00429-010-0262-0 (2010).

10 Calhoun, V. D. *et al.* fMRI activation in a visual-perception task: network of areas detected using the general linear model and independent components analysis. *NeuroImage* **14**, 1080-1088, doi:10.1006/nimg.2001.0921 (2001).

11 Uddin, L. Q. *et al.* Salience network-based classification and prediction of symptom severity in children with autism. *JAMA Psychiatry* **70**, 869-879, doi:10.1001/jamapsychiatry.2013.104 (2013).

12 Greicius, M. D., Srivastava, G., Reiss, A. L. & Menon, V. Default-mode network activity distinguishes Alzheimer's disease from healthy aging: evidence from functional MRI. *Proc Natl Acad Sci U S A* **101**, 4637-4642, doi:10.1073/pnas.0308627101 (2004).

13 Uddin, L. Q., Supekar, K. S., Ryali, S. & Menon, V. Dynamic reconfiguration of structural and functional connectivity across core neurocognitive brain networks with development. *J Neurosci* **31**, 18578-18589, doi:10.1523/JNEUROSCI.4465-11.2011 (2011).

14 Shirer, W. R., Ryali, S., Rykhlevskaia, E., Menon, V. & Greicius, M. D. Decoding subject-driven cognitive states with whole-brain connectivity patterns. *Cereb Cortex* **22**, 158-165, doi:10.1093/cercor/bhr099 (2012).

15 Chen, T., Cai, W., Ryali, S., Supekar, K. & Menon, V. Distinct Global Brain Dynamics and Spatiotemporal Organization of the Salience Network. *PLoS biology* **14**, e1002469, doi:10.1371/journal.pbio.1002469 (2016).

16 Zalesky, A., Fornito, A., Cocchi, L., Gollo, L. L. & Breakspear, M. Time-resolved resting-state brain networks. *Proceedings of the National Academy of Sciences of the United States of America* **111**, 10341-10346, doi:10.1073/pnas.1400181111 (2014).

17 Allen, E. A. *et al.* Tracking whole-brain connectivity dynamics in the resting state. *Cerebral Cortex* **24**, 663-676, doi:10.1093/cercor/bhs352 (2014).

18 Rashid, B. *et al.* Classification of schizophrenia and bipolar patients using static and dynamic resting-state fMRI brain connectivity. *NeuroImage* **134**, 645-657, doi:10.1016/j.neuroimage.2016.04.051 (2016).

19 Allen, E. A. *et al.* Tracking whole-brain connectivity dynamics in the resting state. *Cereb Cortex* **24**, 663-676, doi:10.1093/cercor/bhs352 (2014).

20 Bellec, P., Rosa-Neto, P., Lyttelton, O. C., Benali, H. & Evans, A. C. Multi-level bootstrap analysis of stable clusters in resting-state fMRI. *NeuroImage* **51**, 1126-1139, doi:10.1016/j.neuroimage.2010.02.082 (2010).

21 Menon, V. in *In Brain Mapping: An Encyclopedic Reference* Vol. 2 (ed Arthur W. Toga) 449-459 (Academic Press: Elsevier, 2015).

22 Greicius, M. D., Krasnow, B., Reiss, A. L. & Menon, V. Functional connectivity in the resting brain: a network analysis of the default mode hypothesis. *Proceedings of the National Academy of Sciences of the United States of America* **100**, 253-258, doi:10.1073/pnas.0135058100 (2003).

23 Koul, A., Becchio, C. & Cavallo, A. Cross-Validation Approaches for Replicability in Psychology. *Front Psychol* **9**, 1117, doi:10.3389/fpsyg.2018.01117 (2018).

24 Bzdok, D. & Meyer-Lindenberg, A. Machine Learning for Precision Psychiatry: Opportunities and Challenges. *Biol Psychiatry Cogn Neurosci Neuroimaging* **3**, 223-230, doi:10.1016/j.bpsc.2017.11.007 (2018).

25 Hastie, T., Tibshirani, R. & Friedman, J. H. *The Elements of Statistical Learning: Data Mining, Inference, and Prediction*. (Springer, 2009).
