# Supplementary material for: Aberrant dynamics of cognitive control and motor circuits predict distinct restricted and repetitive behaviors in children with autism
Source: Nat Commun. 2021 Jun 10;12:3537. doi: 10.1038/s41467-021-23822-5 (PMC8192778; doi:10.1038/s41467-021-23822-5)
Supplement: Supplementary file 2 — Reporting Summary [file 41467_2021_23822_MOESM2_ESM.pdf]

nature research

Corresponding author(s): Kaustubh Supekar, Vinod Menon

Last updated by author(s): 04/05/2021

Reporting Summary

Nature Research wishes to improve the reproducibility of the work that we publish. This form provides structure for consistency and transparency in reporting. For further information on Nature Research policies, see our Editorial Policies and the Editorial Policy Checklist.

Statistics

For all statistical analyses, confirm that the following items are present in the figure legend, table legend, main text, or Methods section.

n/a	Confirmed	
☐	☒	The exact sample size (*n*) for each experimental group/condition, given as a discrete number and unit of measurement
☐	☒	A statement on whether measurements were taken from distinct samples or whether the same sample was measured repeatedly
☐	☒	The statistical test(s) used AND whether they are one- or two-sided<br>*Only common tests should be described solely by name; describe more complex techniques in the Methods section.*
☐	☒	A description of all covariates tested
☐	☒	A description of any assumptions or corrections, such as tests of normality and adjustment for multiple comparisons
☐	☒	A full description of the statistical parameters including central tendency (e.g. means) or other basic estimates (e.g. regression coefficient) AND variation (e.g. standard deviation) or associated estimates of uncertainty (e.g. confidence intervals)
☐	☒	For null hypothesis testing, the test statistic (e.g. *F*, *t*, *r*) with confidence intervals, effect sizes, degrees of freedom and *P* value noted<br>*Give P values as exact values whenever suitable.*
☒	☐	For Bayesian analysis, information on the choice of priors and Markov chain Monte Carlo settings
☒	☐	For hierarchical and complex designs, identification of the appropriate level for tests and full reporting of outcomes
☐	☒	Estimates of effect sizes (e.g. Cohen's *d*, Pearson's *r*), indicating how they were calculated

Our web collection on statistics for biologists contains articles on many of the points above.

Software and code

Policy information about availability of computer code

Data collection	Clinical data were collected using standardized questionnaires (Autism Diagnostic Interview - Revised). Imaging (fMRI) data were acquired on a 3T GE scanner.
Data analysis	Clinical data were analyzing using Matlab. Imaging (fMRI) data were preprocessed using SPM8 and analyzed using FSL and Matlab.

For manuscripts utilizing custom algorithms or software that are central to the research but not yet described in published literature, software must be made available to editors and reviewers. We strongly encourage code deposition in a community repository (e.g. GitHub). See the Nature Research guidelines for submitting code & software for further information.

Data

Policy information about availability of data

All manuscripts must include a data availability statement. This statement should provide the following information, where applicable:

- Accession codes, unique identifiers, or web links for publicly available datasets
- A list of figures that have associated raw data
- A description of any restrictions on data availability

Data that support the findings of this study are available from the corresponding authors upon reasonable request.

Field-specific reporting

Life sciences study design

All studies must disclose on these points even when the disclosure is negative.

Sample size	The sample size were determined based on samples from previous neuroimaging studies in children with autism and typically-developing children.
Data exclusions	Children with autism were excluded if they had any history of known genetic, psychiatric, or neurological disorders (e.g., Fragile X syndrome or Tourette's syndrome), or were currently prescribed anti-psychotic medications. Typically-developing children were screened and excluded if they or a first-degree relative had developmental, language, learning, neurological, psychiatric disorders, or psychiatric medication usage, or if the child met the clinical criteria for a childhood disorder on the Child Symptom Inventory – Fourth Edition or Child and Adolescent Symptom Inventory.
Replication	Cross-validation analysis was used to assess replicability of the reported findings.
Randomization	The experimental groups are children with autism and typically-developing children; therefore, randomization is not applicable.
Blinding	Blinding is not relevant to the design of the current study.

Reporting for specific materials, systems and methods

We require information from authors about some types of materials, experimental systems and methods used in many studies. Here, indicate whether each material, system or method listed is relevant to your study. If you are not sure if a list item applies to your research, read the appropriate section before selecting a response.

Materials & experimental systems

n/a	Involved in the study	
☒	☐	Antibodies
☒	☐	Eukaryotic cell lines
☒	☐	Palaeontology and archaeology
☒	☐	Animals and other organisms
☐	☒	Human research participants
☐	☒	Clinical data
☒	☐	Dual use research of concern

Methods

n/a	Involved in the study	
☒	☐	ChIP-seq
☒	☐	Flow cytometry
☐	☒	MRI-based neuroimaging

Human research participants

Policy information about studies involving human research participants

Population characteristics	One hundred and twenty-six children with autism (112 males, 14 females; age: 10.0 ± 1.6 years; IQ: 110 ± 16) participated in the clinical part of the study. Forty-eight children with autism (41 males, 7 females; age: 10.9 ± 1.9 years; IQ: 115 ± 16) and 48 age- and gender-matched typically-developing children (41 males, 7 females; age: 10.9 ± 1.7 years; IQ: 118 ± 11) participated in the imaging part of the study.
Recruitment	Participants were recruited from San Francisco bay area.
Ethics oversight	Written informed consent was obtained from the legal guardian of each child and all study protocols were approved by the Stanford University Institutional Review Board.

Note that full information on the approval of the study protocol must also be provided in the manuscript.

Clinical data

Policy information about clinical studies

All manuscripts should comply with the ICMJEguidelines for publication of clinical research and a completedCONSORT checklist must be included with all submissions.

Clinical trial registration	*Provide the trial registration number from ClinicalTrials.gov or an equivalent agency.*
Study protocol	*Note where the full trial protocol can be accessed OR if not available, explain why.*
Data collection	*Describe the settings and locales of data collection, noting the time periods of recruitment and data collection.*
Outcomes	*Describe how you pre-defined primary and secondary outcome measures and how you assessed these measures.*

Magnetic resonance imaging

Experimental design

Design type: Resting-state fMRI

Design specifications: Participants were instructed to stay awake, keep their eyes closed and try not to move for the duration of the scan.

Behavioral performance measures: *State number and/or type of variables recorded (e.g. correct button press, response time) and what statistics were used to establish that the subjects were performing the task as expected (e.g. mean, range, and/or standard deviation across subjects).*

Acquisition

Imaging type(s): Functional

Field strength: 3T

Sequence & imaging parameters: A total of 29 axial slices (4.0 mm thickness, 0.5 mm skip) parallel to the AC-PC line and covering the whole brain were imaged with a temporal resolution of 2 s using a T2* weighted gradient echo spiral in-out pulse sequence 7 with the following parameters: TR = 2,000 msec, TE = 30 msec, flip angle = 80 degrees, 1 interleave. The field of view was 20 cm, and the matrix size was 64×64, providing an in-plane spatial resolution of 3.125 mm. To reduce blurring and signal loss arising from field in homogeneities, an automated high-order shimming method based on spiral acquisitions was used before acquiring functional MRI scans.

Area of acquisition: Whole brain scan

Diffusion MRI: ☐ Used ☒ Not used

Preprocessing

Preprocessing software: SPM8

Normalization: Non-linear normalization was applied.

Normalization template: MNI152 2mm template was used

Noise and artifact removal: Head motion and signals from white matter and CSF were regressed out.

Volume censoring: *Define your software and/or method and criteria for volume censoring, and state the extent of such censoring.*

Statistical modeling & inference

Model type and settings: *Specify type (mass univariate, multivariate, RSA, predictive, etc.) and describe essential details of the model at the first and second levels (e.g. fixed, random or mixed effects; drift or auto-correlation).*

Effect(s) tested: *Define precise effect in terms of the task or stimulus conditions instead of psychological concepts and indicate whether ANOVA or factorial designs were used.*

Specify type of analysis: ☐ Whole brain ☐ ROI-based ☐ Both

Statistic type for inference (See Eklund et al. 2016): *Specify voxel-wise or cluster-wise and report all relevant parameters for cluster-wise methods.*

Correction: *Describe the type of correction and how it is obtained for multiple comparisons (e.g. FWE, FDR, permutation or Monte Carlo).*

Models & analysis

n/a	Involved in the study
☐	☒ Functional and/or effective connectivity
☒	☐ Graph analysis
☐	☒ Multivariate modeling or predictive analysis

Functional and/or effective connectivity: Sliding-window Pearson correlations were computed

Multivariate modeling and predictive analysis: Mean and variability of CNII/MNII as independent variables and RRB subtype (CI, IS or RM) severity score as dependent variable was used as the input to a linear regression algorithm. Cross-validation analysis was used to assess generalization and reproducibility. Data were divided into five folds. A linear regression model was built/trained using four folds, leaving out one fold. The samples in the leftout fold were then predicted using this trained model, and the predicted values were noted. This procedure was repeated five times, and finally an r(pred, actual) was computed based on the predicted and actual values. r(pred, actual), correlation

between the predicted value of the trained linear regression model and the actual value, was used as a measure of how well the independent variable predicts dependent variable, with r(pred, actual) = 1 being the most accurate prediction model. Finally, the statistical significance of the model was assessed using nonparametric analysis.
